# Repeated Transcranial Magnetic Stimulation for Improving Cognition in Alzheimer Disease: Protocol for an Interim Analysis of a Randomized Controlled Trial

**DOI:** 10.2196/31183

**Published:** 2021-08-09

**Authors:** Zahra Moussavi, Lisa Koski, Paul B Fitzgerald, Colleen Millikin, Brian Lithgow, Mohammad Jafari-Jozani, Xikui Wang

**Affiliations:** 1 Biomedical Engineering Program Faculty of Engineering University of Manitoba Winnipeg, MB Canada; 2 Department of Psychiatry University of Manitoba Winnipeg, MB Canada; 3 McGill University Montreal, QC Canada; 4 Department of Psychiatry Epworth Center for Innovation in Mental Health Monash University Melbourne Australia; 5 Department of Clinical Health Psychology Max Rady College of Medicine University of Manitoba Winnipeg, MB Canada; 6 Department of Statistics & Biomedical Engineering Faculty of Science University of Manitoba Winnipeg, MB Canada; 7 Warren Center for Actuarial Studies and Research The Asper School of Business University of Manitoba Winnipeg, MB Canada

**Keywords:** interim analysis, treatment efficacy, repetitive transcranial magnetic stimulation, Alzheimer disease, double blind, treatment, placebo controlled, randomized

## Abstract

**Background:**

Many clinical trials investigating treatment efficacy require an interim analysis. Recently we have been running a large, multisite, randomized, placebo-controlled, double-blind clinical trial investigating the effect of repetitive transcranial magnetic stimulation (rTMS) treatment for improving or stabilizing the cognition of patients diagnosed with Alzheimer disease.

**Objective:**

The objectives of this paper are to report on recruitment, adherence, and adverse events (AEs) to date, and to describe in detail the protocol for interim analysis of the clinical trial data. The protocol will investigate whether the trial is likely to reach its objectives if continued to the planned maximum sample size.

**Methods:**

The specific requirements of the analytic protocol are to (1) ensure the double-blind nature of the data while doing the analysis, (2) estimate the predictive probabilities of success (PPoSs), (3) estimate the numbers needed to treat, (4) re-estimate the initial required sample size. The initial estimate of sample size was 208. The interim analysis will be based on 150 patients who will be enrolled in the study and finish at least 8 weeks of the study. Our protocol for interim analysis, at the very first stage, is to determine the response rate for each participant to the treatment (either sham or active), while ensuring the double-blind nature of the data. The blinded data will be analyzed by a statistician to investigate the treatment efficacy. We will use Bayesian PPoS to predict the success rate and determine whether the study should continue.

**Results:**

The enrollment has been slowed significantly due to the COVID-19 pandemic and lockdown. Nevertheless, so far 133 participants have been enrolled, while 22 of these have been withdrawn or dropped out for various reasons. In general, rTMS has been found tolerable with no serious AE. Only 2 patients dropped out of the study due to their intolerability to rTMS pulses.

**Conclusions:**

Overall, the study with the same protocol is going as expected with no serious AE or any major protocol deviation.

**Trial Registration:**

ClinicalTrials.gov NCT02908815; https://clinicaltrials.gov/ct2/show/NCT02908815

**International Registered Report Identifier (IRRID):**

DERR1-10.2196/31183

## Introduction

Clinical trials investigating treatment efficacy often incorporate an interim analysis of outcomes. Interim analysis is conducted for a variety of different reasons, which may include detecting unbalanced patterns of adverse events (AEs) in treatment arms with the potential to indicate harm to participants, or determining on statistical grounds whether continuing data collection to the originally planned sample size is likely to provide a definitive answer to the question framed by the primary hypothesis. Recently we have been running a large, multisite, randomized, placebo-controlled, double-blind clinical trial for investigating the effect of repetitive transcranial magnetic stimulation (rTMS) treatment for improving or stabilizing cognition in patients in the mild to moderate stage of Alzheimer disease (AD). All 3 sites are located in urban centers of countries with a socialized health care system (Winnipeg, Montreal, and Melbourne). The details of the protocol are described in [1]. In brief, the study has 2 doses of treatments (either 2 or 4 weeks, 5 days/week) with either an active or a sham coil wherein 1500 pulses at 10 Hz are delivered in 1.5-second trains with 10-second intertrain intervals; the pulses are applied to dorsolateral prefrontal cortex bilaterally. The primary outcome measure is the change in the Alzheimer Disease Assessment Scale-Cognitive Subscale (ADAS-Cog) score from pretreatment to posttreatment. Secondary outcome measures are changes in performance on tests of frontal lobe functioning (Stroop test and verbal fluency) [2], changes in neuropsychiatric symptoms (Neuropsychiatric Inventory–Questionnaire [NPI-Q]), and changes in activities of daily living (Alzheimer Disease Co-operative Study-Activities of Daily Living Inventory [ADCS-ADL]). Tolerability of the intervention is assessed using a modification of the Treatment Satisfaction Questionnaire for Medication (TSQM) [3]. We will assess participants at baseline and 3, 5, 8, 16, and 24 weeks after the start of the intervention. The initial sample size to have a minimum of 80% power level and a significance level of .05 has been estimated as 208 considering 10% dropout.

The goal of the interim analysis is to investigate whether continuing the trial to its planned sample size of 208 is likely to achieve the goal of determining whether active rTMS treatment benefits patients with AD beyond the placebo effect. The objectives of this interim analysis are to (1) ensure the double-blind nature of the data while doing the analysis, (2) estimate the predictive probabilities of success (PPoSs), (3) estimate the numbers needed to treat, (4) re-estimate the initial required sample size.

## Methods

### Overview

The initial estimate of sample size was 208. The interim analysis will be based on 150 patients who will be enrolled in the study and finish at least 8 weeks of the study. Our protocol for interim analysis is explained in detail in the following steps.

### Procedure to Ensure Double-Blind Nature of Data

At the very first stage, the data will be prepared for analysis by a single investigator (ZM) who is aware of group assignment but who does not contribute to the data analysis. This individual will randomly sort and relabel the study participants as P1 (patient 1), P2, P3, etc. The same individual will then randomly sort and label the 3 arms of the intervention (2-week active, 4-week active, and sham) as Group 1, Group 2, and Group 3 before forwarding the data to the statistician. The data will be analyzed by a research assistant and statisticians blind to information about participants contributing to the study, who will also remain unaware of the group (sham versus active) assignment.

### Definition of the Responders

A patient is considered as a (positive) responder to rTMS treatment if s/he meets either one of the 3 criteria below. These criteria are derived based on similar literature monitoring improvement/decline in patients with Alzheimer [4-8]. The literature most commonly suggests a change in ADAS-Cog score from baseline is considered significant (either positive or negative) if the change is 3 points or more from the baseline score. The 3+ score of ADAS-Cog change from baseline (in either positive or negative direction) is considered significant based on studies such as [4]. That study investigated what range of ADAS-Cog change has clinical relevance in a population of 181 patients across 6 months.

Note that in ADAS-Cog and NPI-Q assessments, negative changes from baseline represent improvement, whereas for ADCS-ADL a positive change from baseline implies improvement. In order to avoid confusion, the criteria for responders are written using the term “improvement,” which means a change from baseline toward better cognitive or behavioral function (ie, a positive value for ADCS-ADL and a negative value for ADAS-Cog and NPI-Q).

We define the responders/nonresponders by applying the following criteria. Note that the AND is a logical AND.

- Having 3+ score improvement in the ADAS-Cog score (compared with baseline) at *either* Week 5 *or* Week 8 (*marked positive response*).
- Having a nonsignificant improvement (<3 score) in ADAS-Cog *AND* an improvement or same (ie, improvement score 0) in ADCS-ADL *or* NPI-Q at *either* Week 5 *or* Week 8 (*moderate response*). If the AND part does not hold, then it is considered as a *Small Response*.
- Having a nonsignificant worsening (<3 score) in ADAS-Cog *AND* an improvement (1) score in *both* ADCS-ADL and NPI-Q at *either* Week 5 *or* Week 8 (*small/stabilized response*); otherwise, if the AND part does not hold, the participant is considered as *nonresponsive*.

The above definition of responders is a slightly stricter version of the definitions of responders commonly used in studies to investigate the effect of donepezil (Aricept); for a review, see [5]. It also differs from those studies on donepezil’s efficacy in that the latter outcomes were analyzed at 6 or 12 months after the intervention.

Among the responder groups, we will identify patients with small, moderate, and marked responses, and then estimate the “number needed to treat (NNT)” for each type of response, as NNT is also a measure of the efficacy of the treatment [7].

### Definition of Success

Because rTMS treatment has been suggested as an alternative nonmedication treatment for AD, it makes sense to define its success rate similar to the trials investigating the efficacy of a “standard” medication.

The most commonly used medication for AD is donepezil (Aricept). Several studies have shown significant differences in the number of responders to donepezil versus sham/placebo [4-8]. However, one should also note that the number of nonresponders in all those studies has been much higher than the number of responders. For example, a review of 5 clinical trials [5] of donepezil showed that the ratio of responders versus nonresponders for active treatment was 26/74, whereas the placebo effect response ratio was 14/86. An important meta-analysis [6] of 14 randomized, double-blind, placebo-controlled trials of cholinesterase inhibitors (donepezil, rivastigmine, and galantamine) used in therapeutic doses for at least 12 weeks estimated NNT for different levels of improvement. Their results showed the NNT for 1 additional patient to benefit from the treatment was 7 to achieve stabilization or better, while it was 12 for minimal improvement or better, and 42 for marked improvement. Moreover, the NNT for 1 additional patient to experience an AE was 12. All these values were estimated at the 95% confidence interval.

To guide decision making regarding whether to discontinue or continue the clinical trial until reaching the planned target sample, we will derive predictive probabilities for the study if it continues as opposed to relying only on the *P* values of the analysis at the time of interim analysis.

Based on the above literature [4-8], if the rTMS treatment (*either* dose of the treatment: 2 or 4 weeks) results in *similar or better* outcomes (on average) than those of cholinesterase inhibitor medications as reported in the literature (cited references), and the predictive probabilities are also in favor of similar or better results than those medications after reaching the planned target, then our study should continue; otherwise, the study might be terminated.

### Basic Analysis Details

For the interim analysis, we will use the primary outcome measure (ADAS-Cog) and 2 secondary measures of ADCS-ADL and NPI-Q, which are the most commonly used tests to evaluate improvement or decline of a patient with Alzheimer over time in clinical trials. The changes in these measures compared with baseline will be analyzed.

As the very first step, descriptive basic statistics will be provided to compare the mean and standard deviation of values among the 3 study sites. As the ADAS-Cog, ADCS-ADL, and NPI-Q are all continuous variables, we will use an analysis of covariance (ANCOVA) model to compare the 3 treatment groups (4 and 2 weeks of active and sham).

The models of efficacy will contain covariates for baseline score, treatment effect, and center effect. The parameters for the efficacy as well as futility models are the changes from baseline of the 3 outcome measures among responders (all 3 levels) and nonresponders in each of the 2 active treatment groups versus sham. The standard assumptions on covariance will be tested before running the ANCOVA. If they fail the normality tests, we will use equivalent nonparametric tests (ie, ranked ANCOVA) [9]. The Fisher least significant difference procedure will be used to control for multiple comparisons (responders/nonresponders of each active treatment group) with sham group.

To enroll patients into the study, we use their age and Alzheimer severity measured by the Clinical Dementia Rating Scale sum of boxes score [10] for stratified randomization to the arms of the study. At the interim analysis, and also at the end of the study, demographic variables of age and sex will be investigated using analysis of variance models with factors for treatment and site. Within-group changes in the 3 outcome measures will be analyzed using paired *t* tests. Between-group differences will be investigated by ANCOVA.

We will also investigate the occurrence, if any, of serious AEs that lead to withdrawal of participants from the study in relation to the site and treatment dose.

### Predictive Probabilities of Success

Conditional power is basically the power of the test, that is, the probability to not reject the null hypothesis when it is false. At interim analysis, the conditional power is estimated as the probability of rejecting the null hypothesis of no effect, given a specific alternative hypothesis. PPoSs [11] are weighted averages of the conditional powers across the current probability that each success rate is the true success rate (ie, weighted by the posterior distribution from the existing data). In other words, PPoS is the probability of achieving a successful (significant) result at a future analysis, given the current interim data that have a specific alternative hypothesis. Hence, predictive probabilities are a much more realistic value of predictive trial success than any single estimate of conditional power. The PPoS will be estimated using available statistical software for Bayesian calculation using noninformative prior probabilities. Nevertheless, the following is a series of steps that will be done for PPoS estimation as suggested in [12]:

- At an interim analysis, sample the parameter of interest θ from the current posterior given current data X(θ). The parameter θ is the responses of patients in the study.
- Complete the data set by sampling future samples X(m), observations not yet observed at the interim analysis, from the predictive distribution.
- Use the complete data set to calculate the success criteria (*P* value, posterior probability). If success criteria are met (eg, *P*<.05), the trial is likely to be a success.
- Repeat the first 3 steps for a total of B times (B is an arbitrary but reasonable number defined by the statistician); the PPoS is the proportion of simulated trials that achieve success.

### Confounding Variable (Acetylcholinesterase Inhibitor Medication Effect)

In our study, the majority of patients are on a stable dose of an acetylcholinesterase inhibitor (AChEI) medication. No participant changes or starts an AChEI medication after being enrolled into the study. However, because 35% (47/133) of participants so far are not on any AChEI medication, it should be considered as a confounding variable when analyzing the results.

As the number of participants is still small given the number of independent variables, we will use permutation statistical analysis that tests whether the observations are independent and does not make any assumption about the data’s distribution. If we find the intervention arms unbalanced (statistically) in terms of the number of nonmedicated patients, we will have to adjust our analysis for such a confounding variable.

### The Numbers Needed to Treat (NNTs)

The NNT is another measure to summarize effects of a treatment based on the relative risks. Thus, many clinical trials do calculate the NNT at the end of study or at interim analysis. The NNT for 1 patient to be a responder (either in mild, moderate, or marked response groups) will be calculated by predictive probabilities and method presented by [7].

### Sample Size Re-estimation

At interim analysis for efficacy, a trial can be stopped early by reassessing the sample size based on existing data in case the sample size was overestimated. By contrast, if the sample size initially was underestimated, at the interim analysis, the PPoS can give a better estimation of what sample size is needed for the data to support the study’s hypotheses. We will reassess the sample size at the interim analysis by the method introduced in [8].

## Results

### Current Trial Status

As of May 1, 2021, a total of 523 patients were screened, of whom 133 were enrolled across the 3 sites of the study (62 in Manitoba, 39 in Quebec, and 32 in Australia) and randomized to different arms of the study. Of the 133 participants, 110 have completed the 6-month study period, 1 is waiting to start the treatment (still on hold due to the pandemic), and 22 have withdrawn or discontinued due to different reasons detailed below.

The percentage of the withdrawn cases so far is therefore 16.5% (22/133), which is much higher than our initial 10% estimation. However, partial data for approximately 40% (9/22) of the withdrawn patients can be used for analysis as those were discontinued/withdrawn during the follow-up period after finishing the treatment. The withdrawn cases are categorized into 5 groups. See Table 1 for details and number of withdrawn patients in each category.

**Table 1:** Information on withdrawn patients.

Category	Number	Reasons
Became ineligible during the pandemic	2	They were enrolled before lockdown, but their cognitive function declined rapidly during the lockdown and they became ineligible.
Principal investigator withdrawal—noncompliant	3	One changed medication during the study and 2 developed an illness and were withdrawn by the principal investigator for safety concerns.
Participant withdrawal—changed mind before treatment	7	No particular reason was given.
Participant withdrawal—too much anxiety due to treatment	2	They found the pulses too painful to tolerate.
Unrelated health and family issues	8	Two could not finish treatment due to the pandemic, 2 could not finish treatment due to unrelated health issues, and 4 could not attend all assessment sessions due to unrelated health conditions but their data up to a point can be used.

### Side Effects, Adverse Events, and Tolerability

In this study, at the end of each treatment session, the treatment administrator asks patients via a checklist about any related or nonrelated symptoms and asks them to identify the level of discomfort associated with receiving TMS pulses on a scale of 0-10 as shown in Multimedia Appendix 1. Furthermore, before starting the daily treatment session, the administrator asks the patient whether they had any lingering symptoms from the previous treatment session. In addition, the administrator asks the caregiver on every treatment session if there has been any side effect due to the treatment on the day before. This information is reported to the Data Safety Monitoring Board as well as to the Ethics Board of the study.

The expected side effects of rTMS are scalp pain/sensitivity, leg jerking, toothache, jaw clenching, or eye twitches during the treatment. These symptoms should abate immediately after the end of treatment at each session. If the duration of any of the above symptoms is prolonged, then it is considered as an unexpected side effect.

Other expected side effects include lingering eye twitches, headache, feeling exhausted, or having slight dizziness after the treatment that may last for a few hours. These symptoms are expected to diminish without requiring medication. If they are sustained more than a few hours, they should be listed as unexpected.

Other unexpected side effects that may or may not be related to rTMS treatment include nightmares, prolonged feeling of disorientation, confusion, nausea, fatigue, dizziness, agitation, eye redness, and neck stiffness. Seizure is a rare documented side effect of rTMS among at-risk individuals; for this reason, the screening process is designed to exclude such patients from participating and a protocol for management of an unexpected seizure is in place at each site.

Information about possible side effects is written on the consent forms and explained to each participant and his/her caregiver so they are knowledgeable when signing to provide their informed consent prior to enrollment into the study. AEs, whether expected or unexpected, are managed according to the protocols developed for the Ethics Board at each site. The association of serious AEs, that is, any prolonged side effects beyond a day or any side effect that needed medical intervention, with the treatment protocol is determined by the teams’ physician(s) after consultation with the site PI, patient, and any caregivers involved in the study. Nonserious expected AEs are referred to the site PI for documentation. Nonserious unexpected AEs are referred to the PI, who will consult with the team’s physicians as necessary to determine their association with the treatment protocol. The extensive list of side effects is reported to the Data Safety Monitoring Board of the study as well as to the Ethics Board of the University of Manitoba on a regular basis.

To date, there has been no serious AE. Nevertheless, out of the 133 participants, 89 reported minor typical AE of the rTMS treatment and 12 have reported unexpected AE. The most commonly reported AE has been mild to moderate discomfort and sensitivity to the pulses, with a pain scale of 2-7, while receiving them; however, this reported AE generally reduced over the sessions; most discomfort is reported in the first few sessions of the treatment. The second most commonly reported AE has been fatigue, headache, or both immediately after the treatment, which subsided within a couple of hours without any pain medication. There were also reports of dizziness, disorientation, and nausea after treatment but with much less frequency.

In terms of tolerability, because only 2 of 133 participants withdrew due to finding the rTMS pulses too painful and causing excessive anxiety, we may say overall the participants have tolerated the treatment protocol well.

### Medication Effect on Analysis

In our clinical trial, we enroll patients who are either on a stable dose of an AChEI medication or not taking any cognition-enhancing medication; most importantly they should not change their medication or no-medication status (or dosage, if applicable) during the course of the study (6 months). Thus, we investigated whether the number of nonmedicated patients could have any effect on the interim analysis. Overall, of the 133 participants, 46 were not on cognition-enhancing medication during the time in which they were participating in the study.

Statistical analysis of the data using chi-square and permutation tests of independent variables showed that the mean number of nonmedicated patients did not differ significantly across the 3 arms of the study (*P*>.1). The results so far also showed no significant interaction between time and medication (*P*=.07); in other words, the arms of the study are also stable over the observations made at different times (4 different batches of data, ie, the first, second, third, and fourth group of 33 participants enrolled across the sites), implying that we can expect the same nonsignificant differences in the number of nonmedicated patients among the treatment arms in future. This analysis will be repeated at the interim analysis.

## Discussion

Overall, the study has been going as expected. In general, participants have found the rTMS treatment tolerable and have been compliant with the study protocol; the side effects have been minor and expected in general. Most participants in the sham group have received real treatment at the end of the 6-month study period. Medication can be a confounding variable. Because of the slow enrollment rate of patients with Alzheimer with the strict inclusion/exclusion criteria as in this study, we did not stratify or otherwise control for medication status during the randomization process. To date, 35% (47/133) of patients were not taking cognitive-enhancing medication during participation in the study, and the distribution of such participants does not differ across time or across arms of the trial. Should these results change at the interim analysis, we will adjust for this variable in our statistical analysis.

## Appendix

Multimedia Appendix 1 Interim analysis.

Multimedia Appendix 2 Peer review reports.

## Abbreviations

AChEI: acetylcholinesterase Inhibitor
AD: Alzheimer disease
ADAS-Cog: Alzheimer Disease Assessment Scale-Cognitive Subscale
ADCS-ADL: Alzheimer Disease Co-operative Study-Activities of Daily Living Inventory
ANCOVA: analysis of covariance
NPI-Q: Neuropsychiatric Inventory–Questionnaire
PPoS: predictive probability of success
rTMS: repetitive transcranial magnetic stimulation
TSQM: Treatment Satisfaction Questionnaire for Medication

## References

[1] Moussavi Z, Rutherford G, Lithgow B, Millikin C, Modirrousta M, Mansouri B, Wang X, Omelan C, Fellows L, Fitzgerald P, Koski L (2021). Repeated Transcranial Magnetic Stimulation for Improving Cognition in Patients With Alzheimer Disease: Protocol for a Randomized, Double-Blind, Placebo-Controlled Trial. JMIR Res Protoc.

[2] Stroop Jr (1935). Studies of interference in serial verbal reactions. Journal of Experimental Psychology.

[3] Atkinson MJ, Sinha A, Hass SL, Colman SS, Kumar RN, Brod M, Rowland CR (2004). Validation of a general measure of treatment satisfaction, the Treatment Satisfaction Questionnaire for Medication (TSQM), using a national panel study of chronic disease. Health Qual Life Outcomes.

[4] Schrag A, Schott JM, Alzheimer's Disease Neuroimaging Initiative (2012). What is the clinically relevant change on the ADAS-Cog?. J Neurol Neurosurg Psychiatry.

[5] Mitchell-Box K, Braun KL (2012). Fathers' thoughts on breastfeeding and implications for a theory-based intervention. J Obstet Gynecol Neonatal Nurs.

[6] Lanctôt Krista L, Herrmann Nathan, Yau Kenneth K, Khan Lyla R, Liu Barbara A, LouLou Maysoon M, Einarson Thomas R (2003). Efficacy and safety of cholinesterase inhibitors in Alzheimer's disease: a meta-analysis. CMAJ.

[7] Cook RJ, Sackett DL (1995). The number needed to treat: a clinically useful measure of treatment effect. BMJ.

[8] Posch M, Bauer P (2000). Interim analysis and sample size reassessment. Biometrics.

[9] Cangür Ş, Sungur M, Ankarali H (2018). The methods used in nonparametric covariance analysis. Duzce Medical Journal.

[10] Morris JC (1993). The Clinical Dementia Rating (CDR). Neurology.

[11] Trzaskoma B, Sashegyi A (2007). Predictive probability of success and the assessment of futility in large outcomes trials. J Biopharm Stat.

[12] Saville BR, Connor JT, Ayers GD, Alvarez J (2014). The utility of Bayesian predictive probabilities for interim monitoring of clinical trials. Clin Trials.

