# Supplementary material for: Repeated Transcranial Magnetic Stimulation for Improving Cognition in Alzheimer Disease: Protocol for an Interim Analysis of a Randomized Controlled Trial
Source: JMIR Res Protoc. 2021 Aug 9;10(8):e31183. doi: 10.2196/31183 (PMC8386362; doi:10.2196/31183)
Supplement: Multimedia Appendix 1 [file resprot_v10i8e31183_app1.docx]

**Appendix A: Pain & Side-Effects Assessment Tool**

| Patient:___________________________ |
| --- |
| Visit:______________________________ |
| Date:______________________________ |
| Assessor:__________________________ |

**Pre-rTMS Treatment.**

1. Was there anything unusual or were there any side effects you felt, saw, or noticed yesterday after you went home? **Yes / No**
2. What changes did you notice? (Circle all that apply)

| Sleep | Appetite | Headache | Dizziness | Nausea | Fatigue | Pain |
| --- | --- | --- | --- | --- | --- | --- |

1. If yes, please describe the sensations or symptoms:

|  |
| --- |
|  |
|  |
|  |

1. Are you experiencing any kind of pain or discomfort now? **Yes / No**
2. If yes, please describe the sensations or symptoms:

|  |
| --- |
|  |
|  |
|  |

**“I am going to start now. If you ever feel pain let me know.”**

**During and Post rTMS Treatment.**

1. How was that? Did you feel any pain? **Yes / No**
2. Was the pain somewhere on your head? **Yes / No**

How much did it hurt? __________

**Left / Right**


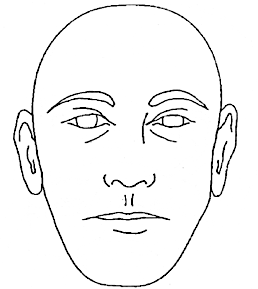

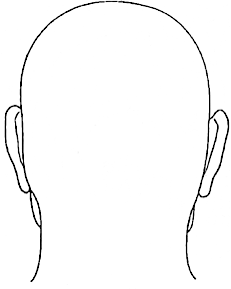

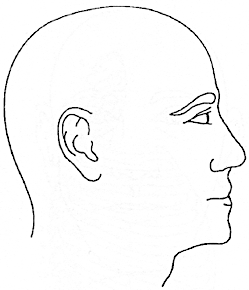

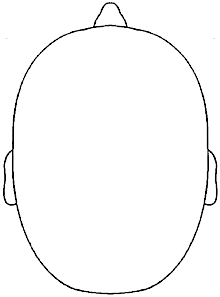


**Fig.** A1

1. Was the pain somewhere on your body? **Yes / No**

**Fig.** A2

1. How much did it hurt? __________


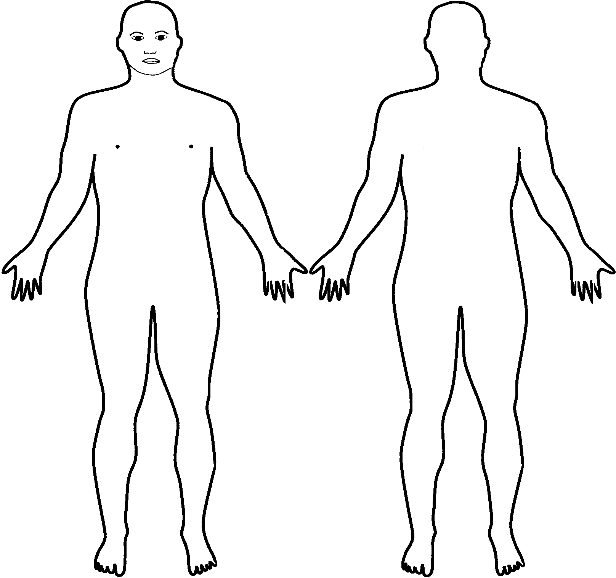

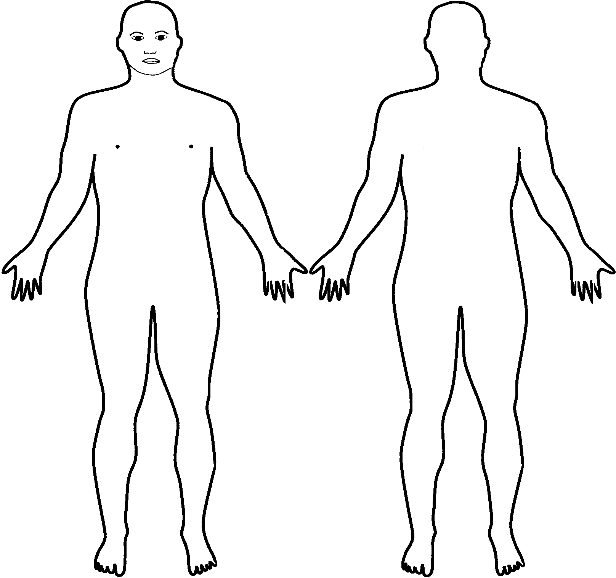


1. Does it still hurt now that it’s stopped? **Yes / No**
2. Notes:

|  |
| --- |
|  |


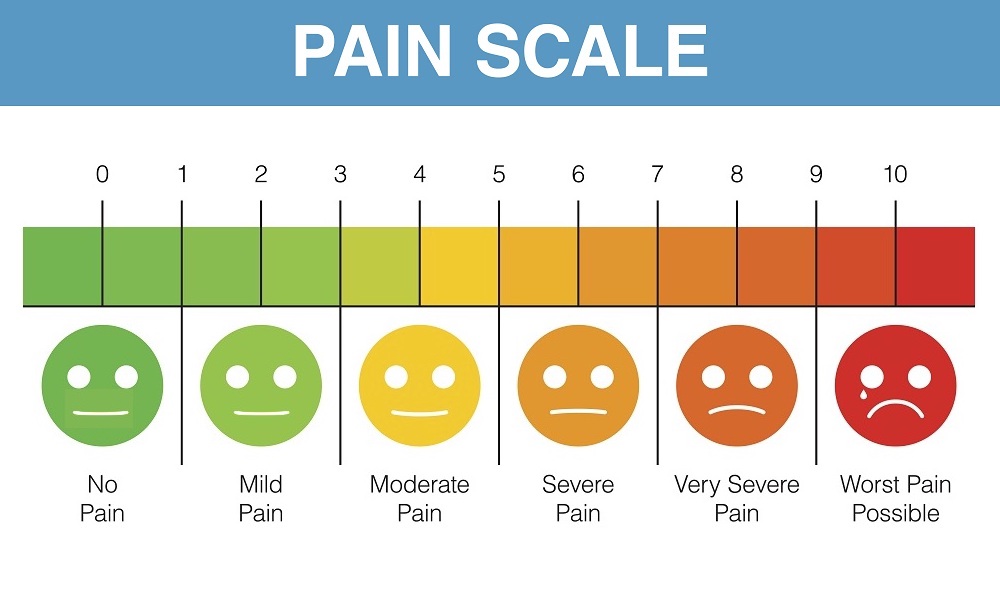


**Fig.** A3
