# Supplementary material for: Repeated Transcranial Magnetic Stimulation for Improving Cognition in Alzheimer Disease: Protocol for an Interim Analysis of a Randomized Controlled Trial
Source: JMIR Res Protoc. 2021 Aug 9;10(8):e31183. doi: 10.2196/31183 (PMC8386362; doi:10.2196/31183)
Supplement: Multimedia Appendix 2 [file resprot_v10i8e31183_app2.pdf]

**Application Number:**	CT140075
**Project Title:**	Investigating the efficacy of high-frequency rTMS treatment for Alzheimer's disease
**Principal Applicant:**	Zahra Kazem-Moussavi

Comments

REVIEWER 1:

1. *Experimental approach:*
The applicants have reasonably addressed all of the methodological issues raised in the review process, including providing a reasonable summary of the strengths and weaknesses of their approach (acknowledging, as they do, that no approach is without limitations). There is clear evidence of input from the right kinds of experts.

2. *Development plan for the therapeutic:*
As above; in addition, feasibility issues are addressed satisfactorily.

3. *Therapeutic impact:*
There are no new issues in this regard. This was regarded favorably in prior reviews.

4. *Team and environment:*
Strengthened adequately.

5. *Budget and milestones:*
none

REVIEWER 2:

1. *Experimental approach:*
Based on the revision process I consider the experimental approach strong.

I would have included one neuroimaging measure (e.g., fMRI; EEG) but authors were asked not to include these measures. I personally hope that the authors, if they will have the opportunity through other funding, will include/develop a sub-project section with surrogate measures of TMS, even if only in a smaller-sample.

2. *Development plan for the therapeutic:*
I believe that the plan is well designed.

3. *Therapeutic impact:*
To date, there are few available therapies for persons suffering of Alzheimer disease, and the results from this project will indeed help to open new prospects, with an important therapeutic impact. So I personally believe that this project is a very good opportunity to adequately evaluate the therapeutic impact of transcranical magnetic stimulation in AD.

4. *Team and environment:*
Overall the team is excellent, it is a very large and rich team involving a large amount of different expertise. This is a good starting point because integrations from scientists of such different background can provide new ways to solve problems brilliantly.

5. *Budget and milestones:*
I'm not able to comment on the budget requested. The milestones are appropriate for the proposed project, they are clear and based on precise outcomes. Yes, the timeline is suitable.

REVIEWER 3:

1. *Experimental approach:*
The experimental approach is excellently suited to answer the research question in all aspects.

2. *Development plan for the therapeutic:*
The results of the study will deliver clear hints about the suitability of rTMS for the treatment of AD and will deliver important information on how to develop optimized intervention protocols.

3. *Therapeutic impact:*
Since no satisfactory therapies for AD do exist at present, the potential therapeutic impact is large.

4. *Team and environment:*
The team and research environment is excellently suited to conduct the trial.

5. *Budget and milestones:*
The requested budget is completely justified and appropriate for the conduction of the project. The milestones are appropriate and provide clear criteria and outcome measures for each tranche of funding. Timelines are demanding, but suitable for the proposed project.

REVIEWER 4:

1. *Experimental approach:*

Our committee and Dr. Moussavi have worked together for more than one year to design a trial that 1) would be sufficiently powered to test the efficacy of rTMS in AD, 2) that would be able to realistically enroll sufficient patients to meet their recruitment goals, 3) that involved persons with sufficient clinical trial experience to develop and institute a realistic recruitment plan, and which 4) tested more than one stimulation paradigm but not so many or in different locations so that other factors would limit drawing conclusions. To Dr. Moussavi's credit, I believe that after several iterations, her experimental desing now meets all of these goals.

My only remaining concern is whether there is evidence that the assessment tools listed in Table 1.a can be given so frequently without having learning effects influence the outcomes. The concern would be whether there could be improved scores due to patients learning components of the exams.

2. *Development plan for the therapeutic:*

No revisions to the plan for development were requested or offered in this second revision.

3. *Therapeutic impact:*

This reviewer continues to feel that rTMS could have a great therapeutic impact if it proves to be efficacious because it has few if any side effects and is easy to administer. The challenge will continue to be the need to get patients to the doctor's office so many days in a row. However, if it proves to be efficacious, then there may be ways to work around this issue, such as home delivery of rTMS.

4. *Team and environment:*

To her credit, Dr. Moussavi has now assembled a team with more clinical experience who will be able to realistically recruit sufficient patients to test the hypotheses offered by this proposal.

5. *Budget and milestones:*

None
